# Identifying the Medical Lethality of Suicide Attempts Using Network Analysis and Deep Learning: Nationwide Study

**DOI:** 10.2196/14500

**Published:** 2020-07-09

**Authors:** Bora Kim, Younghoon Kim, C Hyung Keun Park, Sang Jin Rhee, Young Shin Kim, Bennett L Leventhal, Yong Min Ahn, Hyojung Paik

**Affiliations:** 1 Department of Psychiatry University of California, San Francisco San Francisco, CA United States; 2 Center for Supercomputing Applications Division of Supercomputing Korea Institute of Science and Technology Information (KISTI) Daejeon Republic of Korea; 3 Department of Neuropsychiatry Seoul National University Hospital Seoul Republic of Korea; 4 Department of Psychiatry and Behavioral Science Seoul National University College of Medicine Seoul Republic of Korea

**Keywords:** suicide, deep learning, network, antecedent behaviors

## Abstract

**Background:**

Suicide is one of the leading causes of death among young and middle-aged people. However, little is understood about the behaviors leading up to actual suicide attempts and whether these behaviors are specific to the nature of suicide attempts.

**Objective:**

The goal of this study was to examine the clusters of behaviors antecedent to suicide attempts to determine if they could be used to assess the potential lethality of the attempt. To accomplish this goal, we developed a deep learning model using the relationships among behaviors antecedent to suicide attempts and the attempts themselves.

**Methods:**

This study used data from the Korea National Suicide Survey. We identified 1112 individuals who attempted suicide and completed a psychiatric evaluation in the emergency room. The 15-item Beck Suicide Intent Scale (SIS) was used for assessing antecedent behaviors, and the medical outcomes of the suicide attempts were measured by assessing lethality with the Columbia Suicide Severity Rating Scale (C-SSRS; lethal suicide attempt >3 and nonlethal attempt ≤3).

**Results:**

Using scores from the SIS, individuals who had lethal and nonlethal attempts comprised two different network nodes with the edges representing the relationships among nodes. Among the antecedent behaviors, the conception of a method’s lethality predicted suicidal behaviors with severe medical outcomes. The vectorized relationship values among the elements of antecedent behaviors in our deep learning model (E-GONet) increased performances, such as F1 and area under the precision-recall gain curve (AUPRG), for identifying lethal attempts (up to 3% for F1 and 32% for AUPRG), as compared with other models (mean F1: 0.81 for E-GONet, 0.78 for linear regression, and 0.80 for random forest; mean AUPRG: 0.73 for E-GONet, 0.41 for linear regression, and 0.69 for random forest).

**Conclusions:**

The relationships among behaviors antecedent to suicide attempts can be used to understand the suicidal intent of individuals and help identify the lethality of potential suicide attempts. Such a model may be useful in prioritizing cases for preventive intervention.

## Introduction

Suicide is an important public health epidemic globally. The suicide incidence in the United States has increased in recent years from 10.9/100,000 in 2006 to 13.3/100,000 in 2015 [1], and nearly 45,000 Americans killed themselves in 2016 [2]. The suicide rate in South Korea is the highest among developed countries, and mortality attributable to suicide exceeds that attributable to common diseases, including diabetes, pneumonia, and liver disease [3]. Suicide is a preventable health problem, but effective prevention strategies are lacking because it is a complex issue, and thus, it is difficult for researchers to develop a cause and prediction model [4].

The management of suicide attempts is an urgent clinical problem, and preventing further attempts is particularly important. The risk of suicide has many components, and of these, a previous suicide attempt is among the most important [5,6]. Understanding the nature of suicide attempts and possible associations with subsequent death by suicide may facilitate the design of interventions targeted at specific risk characteristics for particular individuals, thereby increasing clinical effectiveness and reducing morbidity and mortality in this high-risk population.

Suicide attempts are highly heterogeneous and range from a “cry for help” to a nearly lethal attempt with self-mutilation and actual suicide [7]. In the present study, among the outcomes of suicide attempts, we consider the possible medical lethality of attempts as medical consequences, as well as the severity of the physical harm to individuals. Medical lethality as an outcome can be considered the degree of danger to life resulting from a suicide attempt [8]. In addition, most people who attempt suicide will communicate their intent in various forms before they actually attempt suicide [9]. However, it is unclear whether understanding the specific relationships with the behaviors leading up to actual suicide attempts can help to provide guidance for reducing suicide attempts. The relationship between the lethality of a preceding suicide attempt and medical lethality following a subsequent suicide attempt is unknown [10]. Thus, predictive models and explorations of the thought structures of individuals who attempt suicide are still lacking.

We hypothesized that among individuals who attempt suicide, the relationships among their antecedent suicidal thoughts, behaviors, and communications will exhibit specific patterns, thereby allowing us to predict their future risk and lethality. A network model can be employed to conceptualize the complex dynamic systems comprising each interacting symptom [11-13]. Owing to this advantage of network analysis, previous studies have explored the nested interactions among the features of psychopathology [14] or the symptoms of major depressive disorder [15]. The results obtained by network-based analysis can successfully depict multiple nodes as variables, and multiple edges represent the mutual interactions between each pair of variables (ie, nodes). In this study, we employed network analysis to build a model where the nodes represent unique aspects of the expressed suicidal intent and the edges depict the correlations among these nodes.

After determining the relationship values among the antecedent behaviors, we applied deep learning to identify the medical outcomes of subsequent suicide attempts. Deep learning is an emerging machine learning technique for predictive modeling in various applications, which is based on data observations but without domain-specific knowledge. The application of deep learning in the psychiatric field to develop Woebot, a text-based chatbot, has facilitated depression care [16]. However, the success of machine learning-based approaches has been limited in the identification of the central elements of suicidal intents and in prediction modeling based on the information collected by health care providers to meaningfully enhance clinical care. In this study, we employed a network-based method to explore the connections between communicative behaviors prior to suicide attempts with lethal or less lethal outcomes by using data that are routinely collected by physicians. Moreover, to train the complex connections between the antecedent behaviors, our deep learning model utilized the novel relationship values among suicidal intent elements.

## Methods

### Study Sample

**Figure 1:**
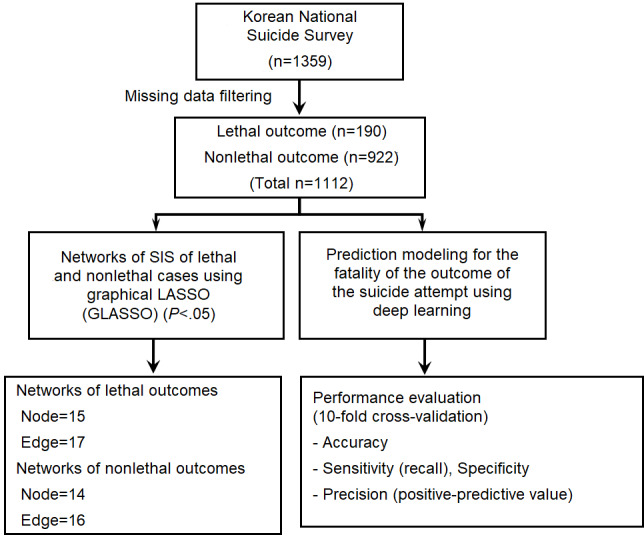
Study overview. C-SSRS: Columbia Suicide Severity Rating Scale; Edge: Association between a pair of nodes based on the weighted correlations according to graphical lasso; Node: Nodes for the measured elements of the SIS and C-SSRS fatality assessment; SIS: Beck Suicide Intent Scale.

We analyzed data obtained from the Korea National Suicide Survey [17], which was a nationwide multicenter study of subjects from two cohorts comprising individuals who attempted suicide and were recruited by retrospective chart review and those who attempted suicide and completed psychiatric evaluations by on-call psychiatric residents. The subjects from the second dataset were used in this study. All individuals who attempted suicide visited the emergency room (ER), and they were evaluated in semistandardized interviews at 17 medical centers across South Korea from May 1, 2013, to November 7, 2013. Deaths in the ER were excluded from the data. Among 1359 individuals who attempted suicide, 1112 were included in the final analysis after excluding missing data from the Columbia Suicide Severity Rating Scale (C-SSRS) and Beck Suicide Intent Scale (SIS) (Figure 1).

### Outcome: Medical Lethality of Suicide Attempts

The outcome of this study involving the medical lethality of suicide attempts was assessed by a clinician and classified based on the “actual lethality or medical damage” using the C-SSRS. The validated Korean version of the C-SSRS was used [18], and lethality was rated as follows: 1, no physical damage or very minor physical damage (eg, surface scratches); 2, minor physical damage (eg, lethargic speech and mild bleeding); 3, moderate physical damage (eg, conscious but sleepy); 4, moderately severe physical damage (eg, comatose with reflexes); 5, severe physical damage (eg, comatose without reflexes); and 6, death [19]. We used a lethality scale with the following two categories: score 3, a less lethal outcome of a suicide attempt and score >3, a lethal outcome of a suicide attempt.

### Suicidal Intents: Suicidal Intent Thoughts, Behaviors, and Communications

The SIS was used to identify the elements of suicidal intent thoughts, behaviors, and communications [20]. The scale contains 15 questions (ie, SIS 1-15), and all of the items are scored on a scale from 0 to 2 for severity, where the total sum of the scores ranges from 0 to 30. In this study, we calibrated the SIS scale from 1 to 3 to calculate the relationships among the features. The SIS comprises the following two parts: objective circumstances of the attempt and the subject’s self-reported intentions and expectations regarding the attempt. The objective factors (SIS 1-8) are as follows: SIS 1, isolation; SIS 2, timing of intervention feasibility; SIS 3, active or passive precautions against discovery or intervention; SIS 4, acting to get help during or after the suicide attempt; SIS 5, final acts in anticipation of death; SIS 6, active preparation for the suicide attempt; SIS 7, suicide note; and SIS 8, overt communication of intent before the suicide attempt. The subjective factors (SIS 9-15) are as follows: SIS 9, alleged purpose of the suicide attempt; SIS 10, expectations of fatality; SIS 11, conception of a method’s lethality; SIS 12, seriousness of the suicide attempt; SIS 13, attitude toward living or dying; SIS 14, conception of medical rescuability; and SIS 15, degree of premeditation.

### Utilization and Reprocessing of Confounders in the ER

We also used clinical data reported from the ER as confounding variables in prediction modeling. In total, 14 confounders were considered, including sex, age, marital status, religion, monthly income, living status, educational level, urbanicity, ER visit date, ER visit on a weekend, ER visit time, admission route, admission transportation, and discharge date. All of the confounders were collected as numerical values, with coded indices or quantitative values as follows: sex (1=male, 2=female), marital status (1=single, 2=married, 3=living together, 4=separated, 5=divorced, 6=widowed), religion (1=Christian, 2=Buddhist, 3=Catholic, 4=Atheist, 5=other), living status (1=living with family, 2=living with somebody, 3=group facilities, 4=living alone), educational level (1=none, 2=elementary school, 3=middle school, 4=high school, 5=undergraduate or higher), ER visit on a weekend (1=yes, 2=no), admission route, admission transportation, and monthly income (self-reported in Korean currency). To represent the date records numerically (ie, year-month-day, ER visit date, and discharge date), we transformed the original date into a decimal year value (eg, 2013-6-30=2013.492). The detailed equation for the numerical transformation of the dates is presented in the supplementary source code [21].

### Relationships Among Suicidal Intent Items

For each pair of 15 suicidal intent items for each individual, we generated three relationship signatures comprising the interaction terms (*I*), harmonized average (*H*), and geometric angle differences using the tangent function (*T*). The definitions of the three relationships between the *ith* and *jth* element of SIS in the *pth* individual (*R*(*I*, *H*, *T*)) are represented by the following equations:


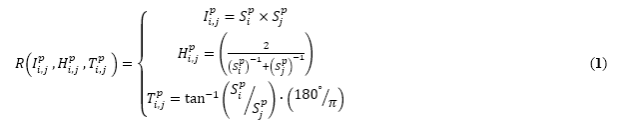


where *S_i_^p^* indicates the *ith* SIS element in the *pth* individual, and *I_i,j_^p^* determines the level of interaction between the *ith* and *jth* SIS elements. To represent the overall intensity in a sensitive manner, we utilized the harmonic mean of a pair of elements (*H_i,j_^p^*). According to the differences in the sequential combination of a pair of elements, such as [*S_i_^p^=2 > S_j_^p^=3*] and [*S_i_^p^=3< S_j_^p^=2*], *T_i,j_^p^* presents a single scalar value for the paired elements.

### Data Analysis

The chi-square test and Student *t* test were performed to compare variables for suicide attempts with lethal and nonlethal medical outcomes.

#### Missing Data Imputation

The k-nearest neighbors algorithm in the R package bnstruct [22] was utilized to impute any missing values (the proportion of missing values in our data was 2.7%).

#### Network Analysis

Network model analysis was performed to build a relational model of lethal and nonlethal suicide attempts. Using the graphical lasso (GLASSO) method, we investigated the weighted correlations between the assessed SIS elements according to attempt fatalities [23]. The network comprised nodes representing the suicidal intent elements, and the edges depicted the relationships among nodes as the medical outcomes of those who had lethal and nonlethal suicide attempts (Figure 1). The statistical significance levels of the GLASSO results were determined using the random 10,000-permutation method (*P*<.05). R [22] and Cytoscape [24] were employed for data analysis and visualization of the results, respectively. The source code was deposited in the GitHub database [21].

#### Machine Learning

Machine learning techniques comprising random forest and linear regression were used. To evaluate the contributions of the relationship scores, we compared the predictive performances of models with or without the relationship features. We utilized TensorFlow [25] to develop our deep learning model called E-GONet. In addition, the feature importance map for the input data of convolutional neural network (CNN) models was generated by DeepExplain with the “Gradient*Input” method [26]. To obtain the feature map, the feature importance scores of all nonlethal cases and all lethal cases were averaged in each fold of 10-fold cross validation sets, and then, the average scores in each fold were averaged into final feature importance scores for nonlethal and lethal cases, respectively.

## Results

### Characteristics of Lethal and Nonlethal Outcomes of Suicide Attempts

Among 1112 individuals who attempted suicide, 190 (17.1%) had suicide attempts categorized as lethal medical outcomes (Table 1). According to the C-SSRS–based fatality of attempt outcomes, we classified the individuals as those who had lethal attempts (n=190, C-SSRS severity 3) and those who had nonlethal attempts (n=922). More male individuals had lethal suicide attempts than nonlethal suicide attempts (107/190, 56.3% vs 357/922, 38.7%). The mean age of those who had lethal suicide attempts was higher than that of those who had nonlethal suicide attempts (47.3 years vs 42.3 years).

The mean total SIS score was higher for those who had lethal suicide attempts than those who had nonlethal suicide attempts (30.23, SD 6.19 vs 25.06, SD 5.61). The total SIS score is the sum of the 15 graded elements of the SIS, such as the scale of isolation from 1 to 3 (complete isolation).

**Table 1:** Demographic characteristics.

Features			Total^a^ (n=1112)		Lethal attempts^a,b^ (n=190)		Nonlethal attempts^a,b^ (n=922)		*P* value
**Sex**									<.001^c^
	Female	647 (58.18)		83 (43.68)		564 (61.17)			
	Male	464 (41.73)		107 (56.32)		357 (38.72)			
	Unknown	1 (0.09)		0 (0.0)		1 (0.11)			
Mean age, years			43.17 (18.2)		47.31 (18.41)		42.31 (18.06)		<.001^d^
**History of suicide attempts**									.88^c^
	No attempt or unknown	763 (68.62)		129 (67.89)		634 (68.76)			
	Previous history	349 (31.38)		61 (32.11)		288 (31.24)			
**C-SSRS^e^ fatality rating for the outcome of an attempt**									
	1: None or minor physical damage	177 (15.92)		N/A^f^		177			
	2: Minor physical damage	387 (34.80)		N/A		387			
	3: Moderate physical damage	358 (32.19)		N/A		358			
	4: Severe physical damage	152 (13.67)		152 (80.00)		N/A			
	5: Very severe physical damage	33 (2.97)		33 (17.37)		N/A			
	6: Death	5 (0.45)		5 (2.63)		N/A			
Mean of the SIS^g^ sum			26.74 (5.91)		30.23 (6.19)		25.06 (5.61)		<.001^d^

^a^Data are presented as n (%), n, or mean (SD).

^b^Fatality scale of the Columbia Suicide Severity Rating Scale (3 for a lethal suicide attempt and <3 for a nonlethal suicide attempt).

^c^Chi-square test between those who had lethal attempts and those who had nonlethal attempts.

^d^*t* test between those who had lethal attempts and those who had nonlethal attempts.

^e^C-SSRS: Columbia Suicide Severity Rating Scale.

^f^N/A: not applicable.

^g^SIS: Beck Suicide Intent Scale.

### Network Model Based on Suicidal Intent Elements

Using GLASSO, we determined the weighted correlations among the assessed SIS elements according to suicide attempt fatalities [23]. We constructed two networks comprising nodes representing the SIS elements (eg, degree of isolation) and edges depicting the relationships among nodes, which indicated the distinct relationships between the elements of suicide in those who had lethal and those who had nonlethal suicide attempts (Figure 1). The statistical significance of the correlations assessed among the SIS elements using GLASSO were determined based on random distributions of the SIS elements (*P*<.05 for random distributions). Finally, we represented the distinct relationships between the suicidal intents of those who attempted suicide (lethal and nonlethal cases).

**Figure 2:**
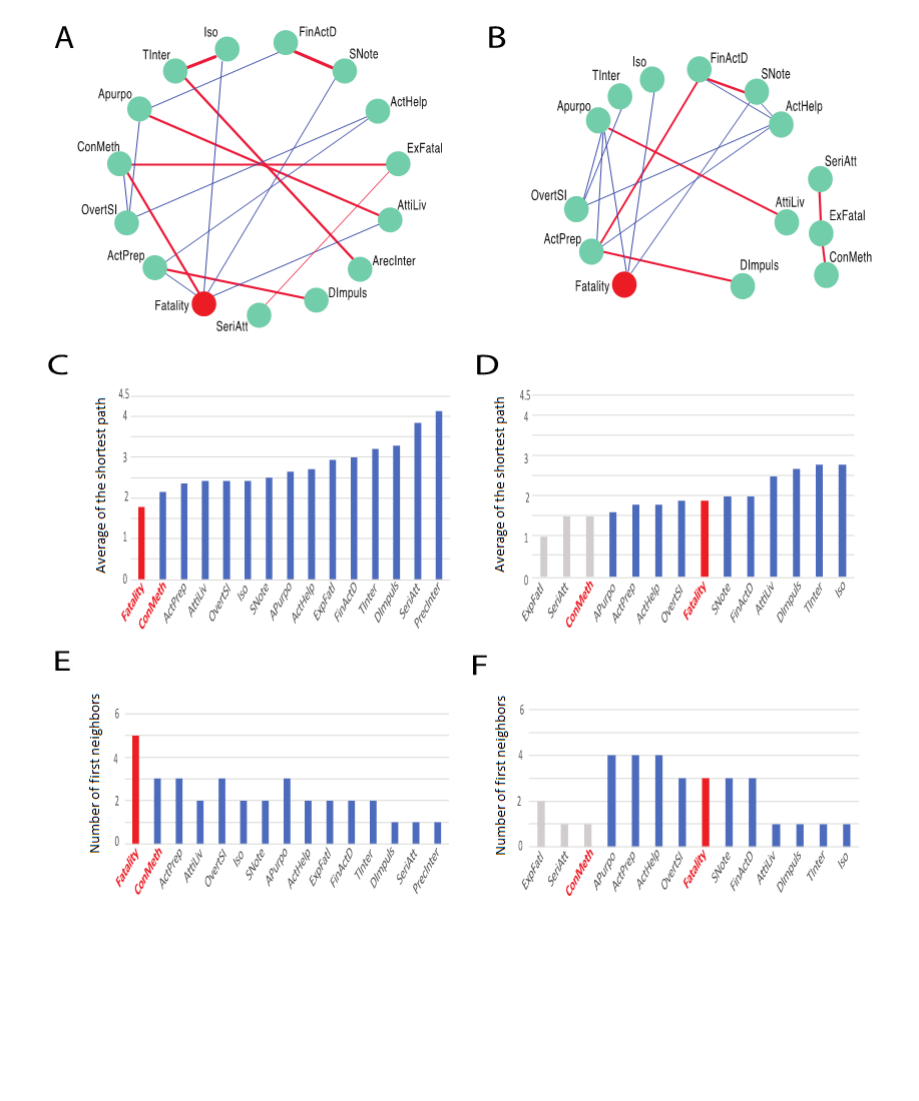
Network structures obtained for lethal and nonlethal outcomes. (A, B) Networks obtained for lethal (A; n=190, C-SSRS fatality ≥3) and nonlethal (B; n=922, C-SSRS fatality <3) cases. Each node represents the SIS element (green circles) and assessed fatality of the attempt using the C-SSRS score (red circles). The linked edges indicate strong relationships between nodes based on the weighted correlations obtained by graphical lasso (GLASSO) (*P*<.05). The red edges represent positive relationships, and the blue edges represent negative relationships. The cyan circular nodes indicate SIS elements, and the red circular nodes indicate the C-SSRS fatality scores for suicide attempts. (C-F) Bar charts showing the topological properties of the networks obtained for lethal (C, E) and nonlethal cases (D, F). The SIS elements were as follows: isolation (Iso), time intervention feasibility (Tinter), active or passive precautions against discovery intervention (ArecInter), acting to get help (ActHelp), final acts in anticipation of death (FinActD), active preparation for attempt (ActPrep), suicide note (SNote), overt communication of suicidal intent (OvertSI), alleged purpose of attempt (Apurpo), expectation of fatality (ExFatal), conception of method lethality (ConMeth), seriousness of attempt (SeriAtt), attitude toward living or dying (AttiLiv), conception of medical rescuability (ConResc), and degree of premeditation impulsiveness (Dimpuls). C-SSRS: Columbia Suicide Severity Rating Scale; SIS: Beck Suicide Intent Scale.

Fifteen nodes for suicidal intents were linked via 17 edges for lethal suicide attempts (n=190) (Figure 2A). Among the 922 individuals who had nonlethal suicide attempts, there were 16 relationships (ie, edges) among 14 suicidal intent elements (ie, nodes) in the nonlethal suicide attempts (Figure 2B). The edges between nodes represent the positive or negative relationships between nodes based on the GLASSO results (*P*<.05 for edges based on a random distribution). Among individuals who had lethal attempts, the fatal outcomes of suicide attempts were more tightly linked (ie, associated) with the suicidal intents compared with those who had nonlethal attempts, and the nodes for the SIS elements were loosely connected or separated in those who had nonlethal attempts (Figure 2A and B). Thus, the close connectedness of the suicidal intent elements, including the concept of lethality of the method, was stronger among those who had lethal attempts.

The topological properties of the network, such as the central node that had the largest number of relationships with other nodes and the average of the shortest paths (ie, degree of centrality), were employed to determine the central suicidal intent elements for the lethal and nonlethal attempts (Figure 2C-F). Among the lethal suicide attempts, the *fatality of suicide attempt* (“Fatality” node) and the *conception of a suicide method’s lethality* (“ConMeth” node) were strongly associated with other suicidal intent elements (Figure 2E). In Figure 2C, the y-axis denotes the average shortest path, which was a bottleneck and a central node, and the “ConMeth” node was ranked highly among those who had lethal attempts (Figure 2C). However, the *alleged purpose of suicide attempt* (“APurpo” node), including “to manipulate the environment,” was a crucial intent element in the minds of those who had nonlethal attempts (Figure 2D and F). Moreover, among those who had nonlethal attempts, suicide method-related features (eg, the conception of a method’s lethality and the expectation of fatality), which are closely linked to lethality among those who had lethal attempts, were completely disconnected from the other suicidal intent nodes (Figure 2D and F).

Thus, we elucidated the relationships between the suicidal intent elements in those who had lethal and those who had nonlethal suicide attempts. The *conception of a method’s lethality* (“ConMeth” node) was a central suicidal intent element, which was clearly related to lethal suicide attempts, and it was connected with the initiation of attempts, such as the nodes for *expectation of fatality* (“ExFatal” node) and *seriousness of attempt* (“SeriAtt” node).

### Predictive Model for Medical Lethality of Suicide Attempts Based on Deep Learning: E-GONet

**Figure 3:**
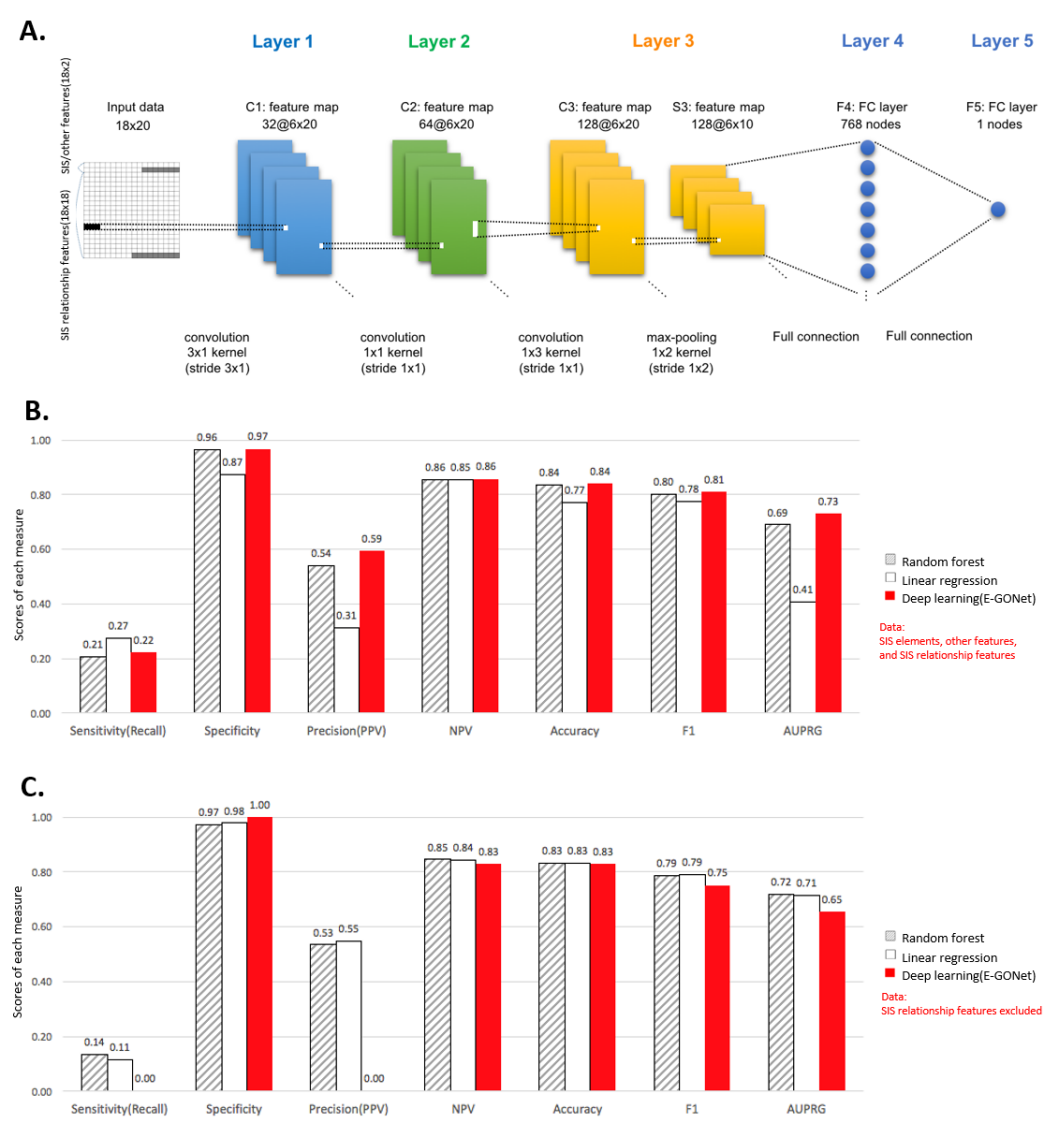
Construction of E-GONet and performance evaluation. (A) Structure of E-GONet based on a convolutional neural network model. The input data format was 18 × 20 (row × column), which comprised SIS or other features and SIS relationship features. The SIS or other features comprised 29 features (15 SIS features and 14 observations collected by emergency rooms) and seven blanks with all zero values (gray). The SIS relationship features comprised 315 features (105 relationships × three types) and nine blanks. E-GONet has three convolutional layers and two fully connected layers. TensorFlow 1.8.0 was used for the implementation. (B) Mean performance based on 10-fold cross-validation using all of the implemented features in A. The red bar shows the average performance of E-GONet with the 10-fold cross-validation set. (C) Mean performance using the data set without SIS relationship features. AUPRG: area under the precision-recall gain curve; F1: weighted-F1 score; NPV: negative-predictive value; PPV: positive-predictive value; SIS: Beck Suicide Intent Scale.

Owing to the structural differences in the networks of antecedent behaviors according to the lethal or nonlethal outcomes of the suicide attempts, we generated three relationship signatures for each pair of SIS elements comprising the interaction terms (*I*), harmonized average (*H*), and geometric angle differences using the tangent function (*T*). In addition to the 15 SIS elements and 14 types of clinically reported data collected by the ER, including age, admission date, and living status, three relationship signatures were prepared for all possible SIS combinations for each individual. We represented the pairs of SIS elements as specific numeric values, and 315 relationship features were obtained among the 105 combinations of SIS elements for an individual. We built E-GONet based on a CNN, which is a subclass of deep learning. The overall structure of E-GONet comprises input and output layers, as well as multiple hidden layers (convolutional layers, pooling layers, and fully connected layers). The schematic structure of each layer is shown in Figure 3A. Multimedia Appendix 1 and Multimedia Appendix 2 describe the detailed structures of the E-GONet model.

### Evaluation of E-GONet for Identifying the Medical Lethality of Suicide

Figure 3B shows the results obtained from the performance evaluation. We evaluated the predictive performance of the E-GONet model by 10-fold cross-validation. We used the same dataset for the performance comparison with E-GONet and to establish two prediction models with linear regression and random forest (ie, an aggregated decision tree model) [27] methods.

E-GONet performed better than the linear regression and random forest methods (E-GONet increased the F1 score up to 3.4%, and the mean increase in the F1 score was 2.1%; E-GONet also increased the area under the precision-recall gain curve [AUPRG] up to 32.1%, and the mean increase in the AUPRG was 18.1%) [28]. Besides, the positive-predictive value (PPV; precision) comprising the rate of correctly identifying lethal attempts was highest with the E-GONet predictions (0.59). As generally noted in the clinical field, our dataset was relatively imbalanced (lethal 190, nonlethal 922). In analysis involving imbalanced data, sensitivity, PPV, F1, and AUPRG have been used for performance evaluation instead of specificity, negative-predictive value, and accuracy.

The analysis of the contribution of learning features (Multimedia Appendix 3 and Multimedia Appendix 4) showed that most of the contributions of confounding variables (such as level of education) were negligible for the predictive performance of E-GONet. However, the relationship signatures between the SIS element pairs contributed greatly to the superior performance of the E-GONet model (the values of *R*(*I*, *H*, *T*)). As depicted in Multimedia Appendix 3 and Multimedia Appendix 4, the saliency heatmaps of our model highlighted the contribution of SIS relationship features. The first two rows of the feature importance matrixes were the confounders (age, sex, income levels, etc) and SIS elements (SIS 1-15). Out of those features, only age and income level contributed to E-GONet training. On the other hand, as presented in the figures, the developed relationship features of SIS elements were more important for CNN model training. Interestingly, out of all relationships among SIS elements, the relationship with SIS element 11 (conception of a suicide method’s lethality) was the biggest contributor to predictions. This is highly consistent with the network modeling of SIS elements in Figure 2.

Moreover, Figure 3C shows the evaluations of the performance of the models established without the SIS-based relationship features. The predictive models based on linear regression and random forest exhibited similar or lower performance after introducing the SIS-based relationship features, because these classical methods could not patternize the relationship features of suicide elements. However, E-GONet trained and improved performance via the vectorized relationships in high-dimensional spaces. As a result, the relationships between SIS elements increased the performance of the E-GONet model by 60% in precision, 6% in F1, and 8% in AUPRG (precision [without relationship/with relationship]=0.0/0.59, F1=0.75/0.81, and AUPRG=0.65/0.73). The full spectrum of the AUPRG displayed the training and fitting process of our deep learning approach in a very detailed manner (Multimedia Appendix 5). In Multimedia Appendix 6, the standard deviations of AUPRGs are presented via 100 trials of 10-fold cross-validation settings.

Therefore, identifying the lethality of attempt outcomes is feasible with deep learning through the major contributions of the relationships among SIS elements (ie, mutual interactions of antecedent behaviors). To allow the use of our method in clinics, we have made all of the source codes for the analytics available via the internet, including the network-based analytics, E-GONet model, and data preprocessing methods [21].

## Discussion

Using network analysis, we elucidated the relationships among antecedent behaviors prior to suicide attempts, where we identified the unique patterns associated with both lethal and nonlethal medical outcomes of suicide attempts. These findings allowed us to interpret the behaviors before lethal suicide attempts, thereby helping us to systematically investigate the interactions and connections among the behaviors that result in lethal suicide attempts. In particular, suicide attempts with lethal medical outcomes were associated with clear concepts regarding the likely fatality of the methods applied. In addition, behaviors, such as isolation at the time of suicide attempts, were strongly associated with the expected intervention time and the possibility of being discovered by other people. Among nonlethal suicide attempts, the suggested aim of suicide was a central factor among suicidal intent elements. Thus, lethal suicide attempts involved clear notions regarding the success of suicide, whereas nonlethal attempts were focused on the achievement of suicide attempts per se. In addition, prediction based on deep learning performed better after introducing the relationship signatures among the suicidal intent elements (60% increase in precision, 6% increase in the F1 score, and 8% increase in the AUPRG). Based on the analysis of feature contributions, we conclude that training of the relationships among SIS elements, especially isolation and the conception of a method’s lethality, strongly ameliorated the deep learning performance. To the best of our knowledge, this is the first study to successfully discern the differences in mutual interactions among antecedent communicative behaviors prior to suicide attempts by those who had lethal and those who had nonlethal attempts, in which our novel method employed relational signatures to facilitate deep learning–based predictions.

In this study, we found that suicide attempts in individuals who had information about suicide methods and who anticipated fatality before attempting suicide had more lethal consequences. Our previous study showed that suicide methods are highly associated with subsequent suicide-related death [29]. Based on these results, we can infer that possessing information about suicide methods and their severity will affect suicide attempts and the consequent lethal outcome. Information about suicide methods can be found easily via the internet, and previous studies have shown that online searches for suicide-related terms are positively associated with intentional self-injury and death due to suicide [30,31]. In addition, we need to consider that suicide methods are subject to cultural differences. For example, suicide methods employed in the United States are predominantly related to firearms and the suicide rate is related to the gun possession rate by state [32]. By contrast, gun usage is very rare in South Korea because of the legal regulations related to gun possession [33]. However, the use of pesticides is fairly prevalent in suicide attempts in Korea, especially in rural areas and among elderly individuals who attempt suicide [17]. According to our results, we believe that restricting the accessibility of information regarding suicide methods is essential for suicide prevention, and cultural differences should be considered.

In previous studies, a machine learning algorithm trained with the longitudinal electronic health records of patients reliably predicted suicidal behavior [34] and actual suicide among US Army soldiers [35]. Linguistic-driven models that use the text in clinical notes have also been explored, but they lack sufficient accuracy (approximately 65%) [36]. In the future, machine learning based on medical big data may become a ubiquitous component of clinical research and practice, which is a prospect that some find uncomfortable [37]. This study was based on three components comprising psychiatric physicians, data scientists, and a sophisticated computational infrastructure (KAT GPU Cluster System, Intel Xeon Ivy Bridge, 2.50 GHz 10 Cores; NVIDIA Tesla V100). However, the contributions of relationship features to the precise fatality predictions demonstrate that insights from physicians, including our hypothesis (ie, interactions among SIS elements were useful), as well as communication with the algorithm developer, are essential for innovative digital health development and precision medicine.

We have developed new approaches to investigate the characteristics of suicide attempts; however, this study had several limitations. First, the study sample did not represent the whole population of individuals who attempt suicide, as the 17 medical centers were located in specific urban areas of South Korea. The sample only included individuals who attempted suicide and came to the emergency centers [17]. In addition, the characteristics of suicide attempts differ among cultures. However, despite the limitations of the sample, the 1359 individuals who had suicide attempts comprised a large number of those who were assessed by a clinician shortly after their suicide attempts. The 17 medical centers were selected based on their enrollment in the National Emergency Department Information System, which is a government-managed nationwide registration system [38]. Lastly, E-GONet may have additional costs for learning and operating the deep learning model, as a deep learning model would require a more specialized facility with systems like a GPU system. Thus, cost-effectiveness and streamlined operation are the next milestones for deep learning–based approaches. For example, the world’s best artificial intelligence model AlphaGo has a cost of US $35 million. This is much higher than the cost of a single human Go player per game.

As is usually noted in the clinical field, our data were relatively imbalanced (lethal, n=190; nonlethal, n=922). In order to appropriately analyze the data, we tried to use data resampling approaches. We applied an over-sampling method, an under-sampling method, and the synthetic minority over-sampling technique (SMOTE), but these methods did not improve the results. Therefore, we did not apply resampling approaches to our analysis. In addition, since resampling methods could not improve the results, it is expected that applying cost-sensitive loss functions will also not change the results.

In this study, we performed binary classification based on a lethality threshold (lethal >3, nonlethal ≤3). If we perform more fine-grained classification (ie, predict the exact C-SSRS grade), we could obtain more information for tailored care in clinics. However, as depicted in Table 1, the outcome of suicide attempts (C-SSRS grade) can be classified into six levels. Among the six levels, levels 5 and 6 involved very limited numbers of individuals who attempted suicide. Thus, we can only build a regression model for minor physical damage (ie, C-SSRS grades 1, 2, and 3) and severe damage (C-SSRS grade 4). However, because the number of individuals in C-SSRS grade 4 is limited compared with minor damage cases, the regression model for severe damage may not be well developed. Therefore, more fine-grained classification is not appropriate in this study. However, binary classification can provide clinically meaningful information. Regardless of our study design, the fine-grained identification of suicide attempts can be a guideline for further studies.

Two conclusions can be drawn regarding the originality of this study. First, effective management strategies can be provided for the care of individuals who attempt suicide, as individuals with lethal outcomes had expectations regarding the fatality of their suicide attempts and they made great preparations before their attempts so that they would not be found. Second, the enhanced performance of deep learning prediction shows that preprocessing the relationships in patient data using nonlinear transformation (ie, the interaction terms between SIS elements) can help the machine learning process understand the information embedded in clinical practice and employ it to make effective inferences.

The findings of this study may help public health officials and clinicians to identify the profiles of individuals at high risk of recurrent suicide attempts and may facilitate the development of efficient and effective suicide prevention programs.

## Appendix

Multimedia Appendix 1 Structure of E-GONet, which is a convolutional neural network model.

Multimedia Appendix 2 Structure of E-GONet (without relationship features).

Multimedia Appendix 3 Importance of the utilized features for the training of E-GONet.

Multimedia Appendix 4 Detailed contribution of the Beck Suicide Intent Scale relationship features.

Multimedia Appendix 5 Precision-recall gain curves for random forest, linear regression, and E-GONet (with relationship features).

Multimedia Appendix 6 Performance of the area under the precision-recall gain curve.

## Abbreviations

AUPRG: area under the precision-recall gain curve
CNN: convolutional neural network
C-SSRS: Columbia Suicide Severity Rating Scale
ER: emergency room
GLASSO: graphical lasso
PPV: positive-predictive value
SIS: Beck Suicide Intent Scale
