# Supplementary figures and images for: Identifying the Medical Lethality of Suicide Attempts Using Network Analysis and Deep Learning: Nationwide Study
Source: JMIR Med Inform. 2020 Jul 9;8(7):e14500. doi: 10.2196/14500 (PMC7380907; doi:10.2196/14500)

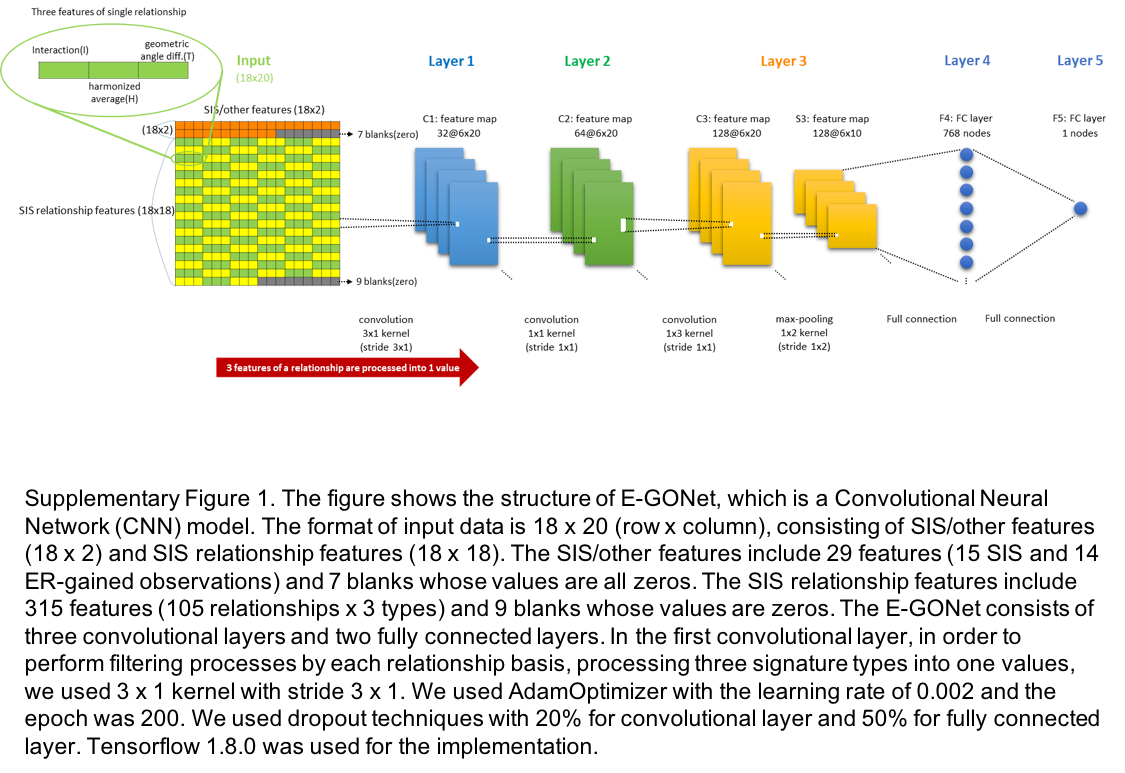

Supplement: Multimedia Appendix 1 [file medinform_v8i7e14500_app1.png]

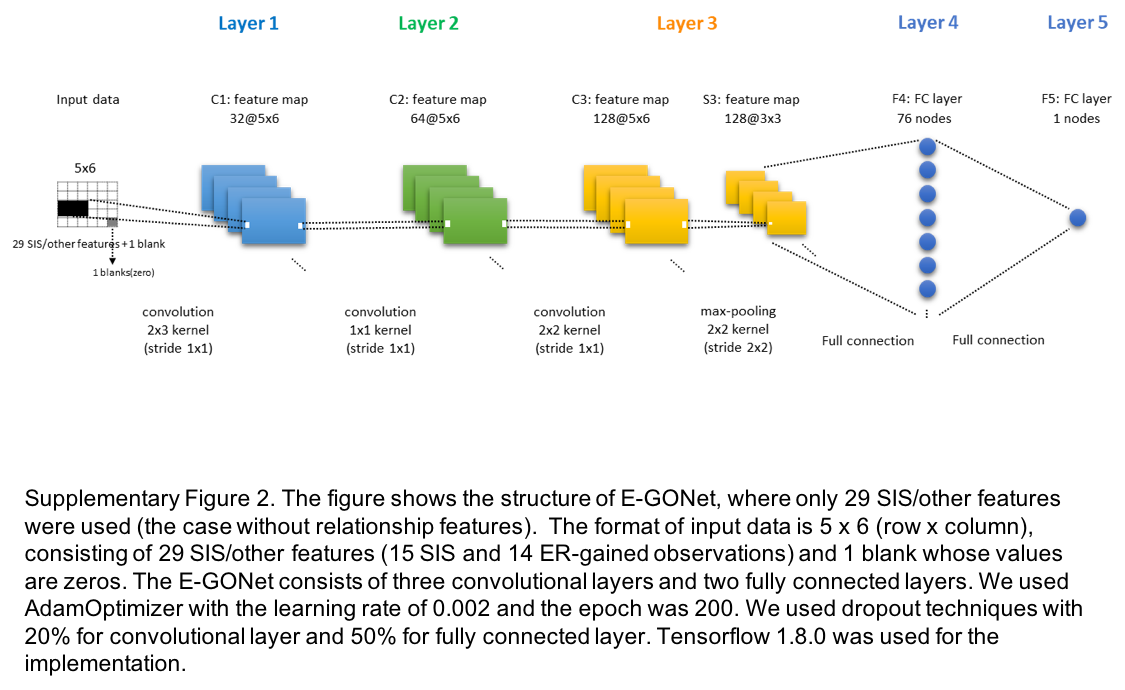

Supplement: Multimedia Appendix 2 [file medinform_v8i7e14500_app2.png]

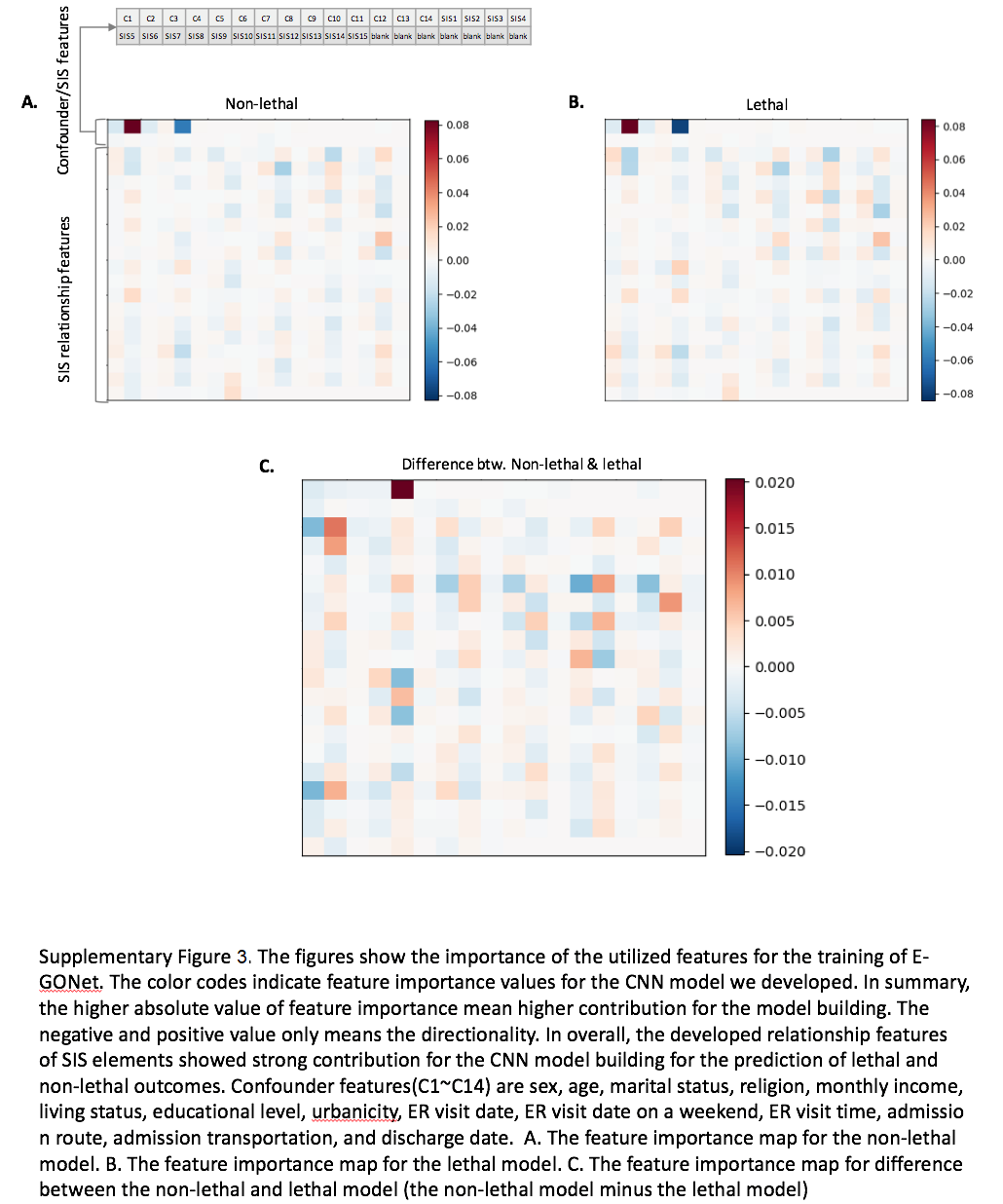

Supplement: Multimedia Appendix 3 [file medinform_v8i7e14500_app3.png]

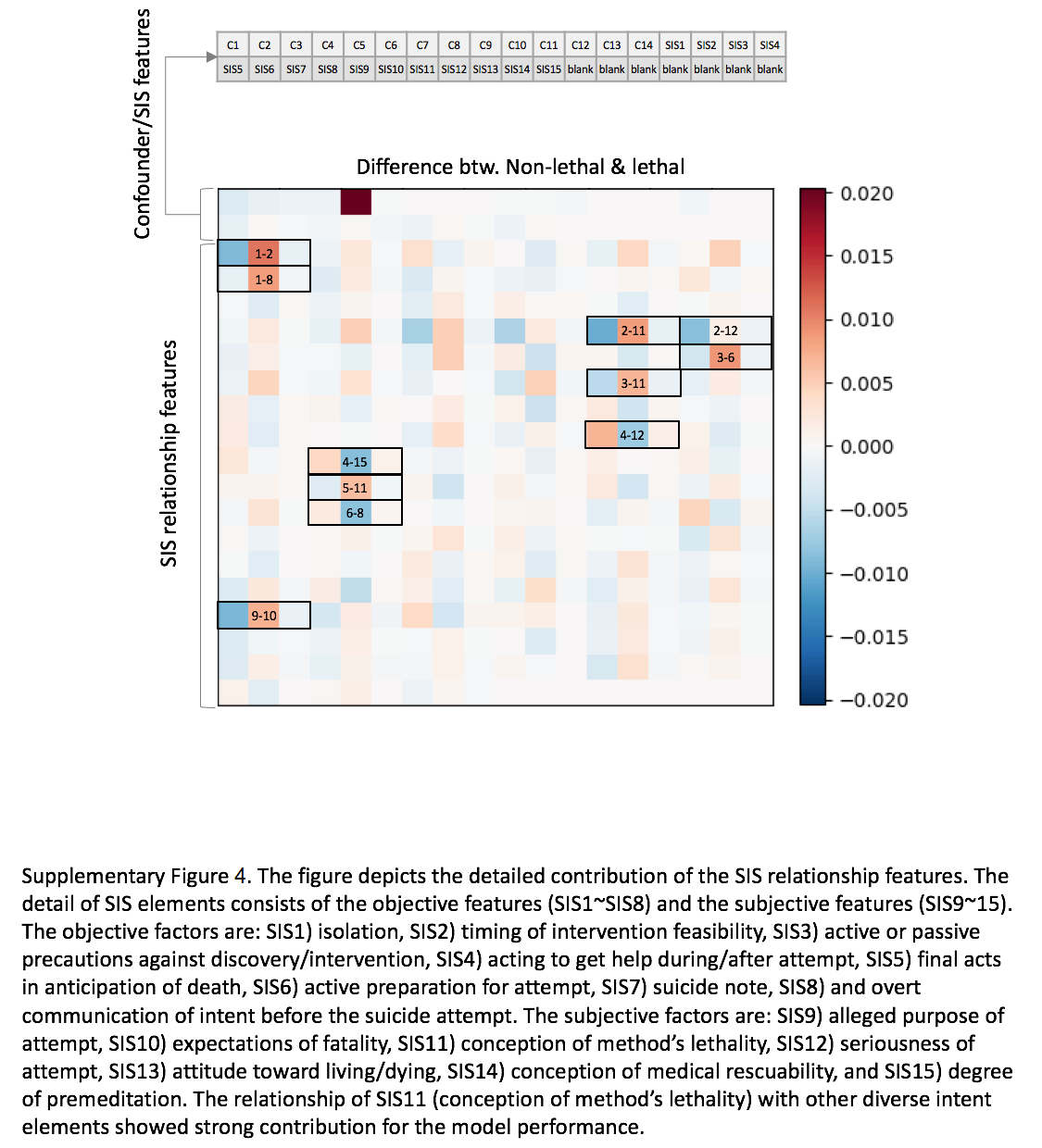

Supplement: Multimedia Appendix 4 [file medinform_v8i7e14500_app4.png]

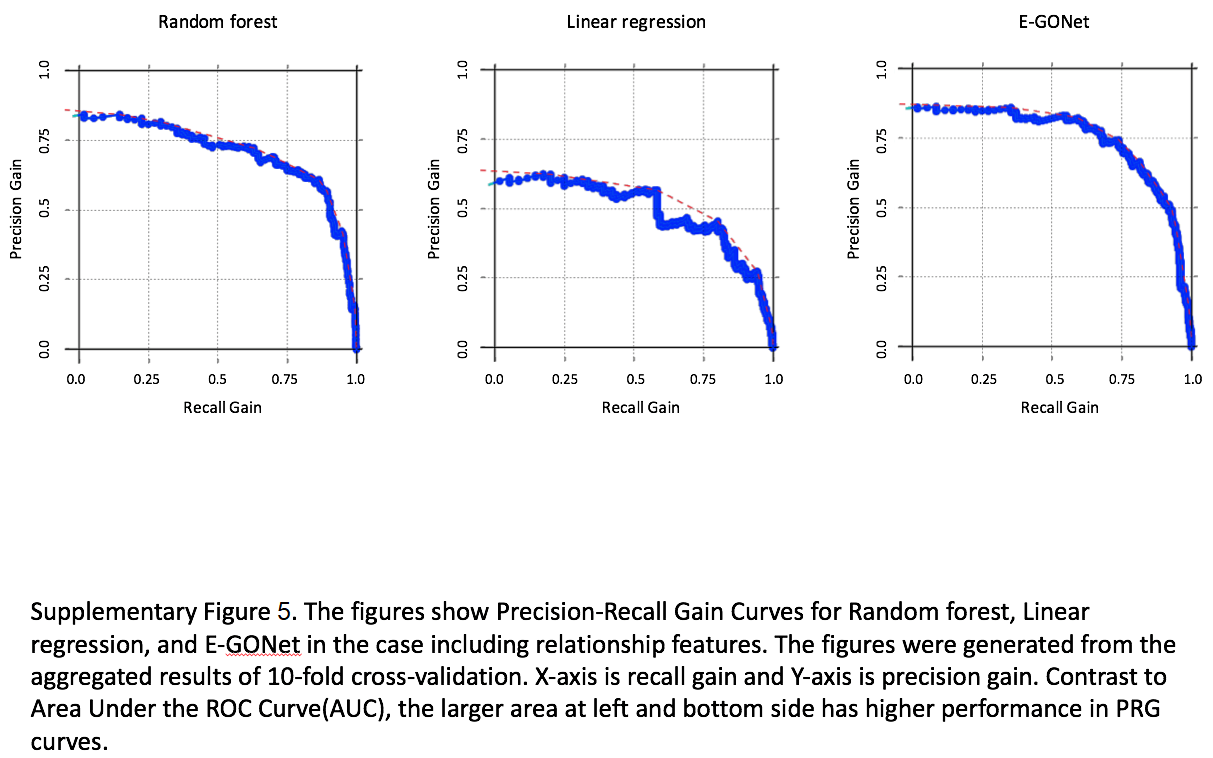

Supplement: Multimedia Appendix 5 [file medinform_v8i7e14500_app5.png]

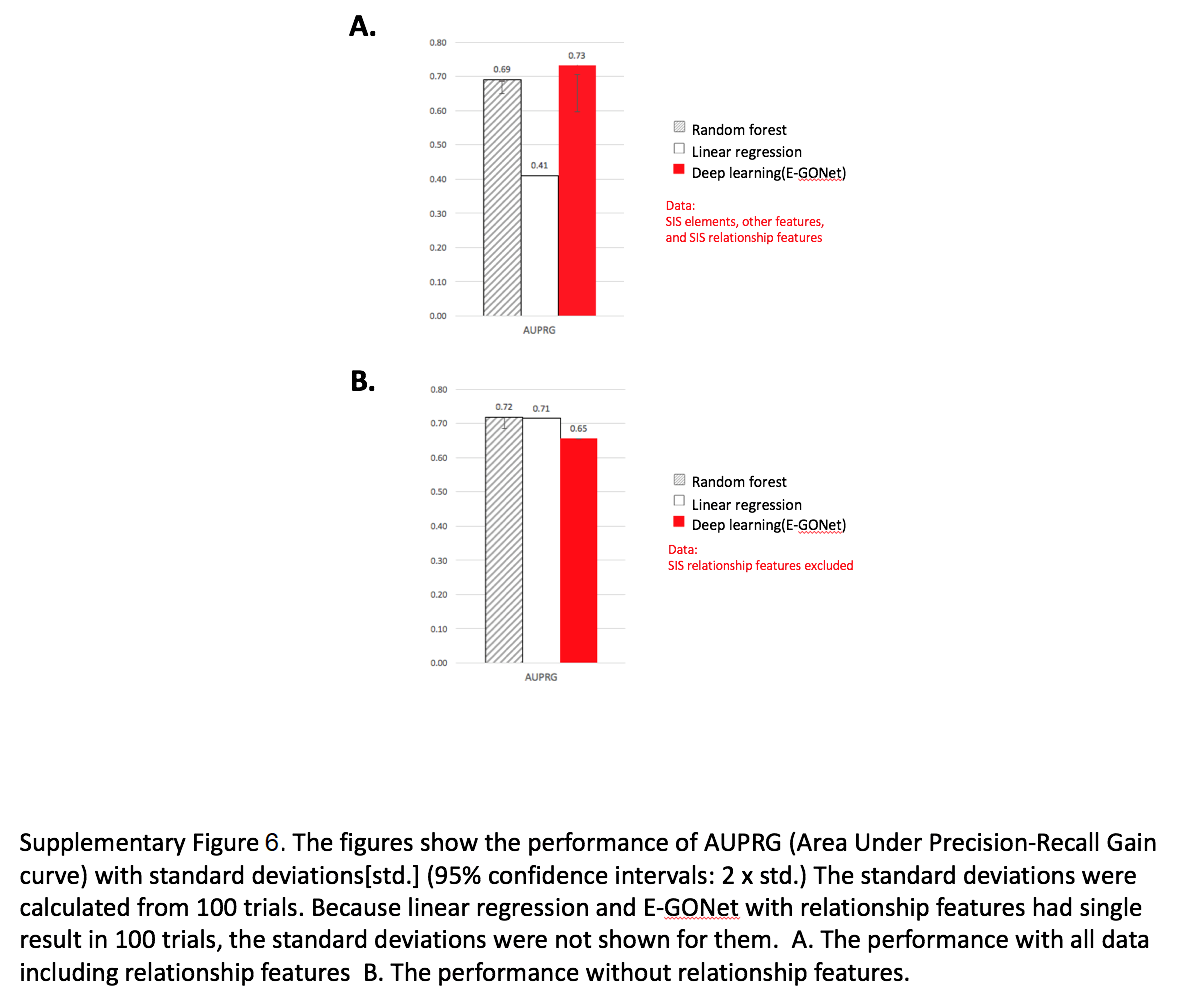

Supplement: Multimedia Appendix 6 [file medinform_v8i7e14500_app6.png]
